# Bacterial Transformation of Aromatic Monomers in Softwood Black Liquor

**DOI:** 10.3389/fmicb.2021.735000

**Published:** 2021-09-10

**Authors:** Laura E. Navas, Gara Dexter, Jie Liu, David Levy-Booth, MiJung Cho, Soo-Kyeong Jang, Shawn D. Mansfield, Scott Renneckar, William W. Mohn, Lindsay D. Eltis

**Affiliations:** ^1^Department of Microbiology and Immunology, Life Sciences Institute, BioProducts Institute, The University of British Columbia, Vancouver, BC, Canada; ^2^Department of Wood Science, BioProducts Institute, The University of British Columbia, Vancouver, BC, Canada

**Keywords:** aromatic compound, bacterial catabolism, lignin, acetovanillone, *Rhodococcus*

## Abstract

The valorization of lignin, a major component of plant-derived biomass, is essential to sustainable biorefining. We identified the major monoaromatic compounds present in black liquor, a lignin-rich stream generated in the kraft pulping process, and investigated their bacterial transformation. Among tested solvents, acetone extracted the greatest amount of monoaromatic compounds from softwood black liquor, with guaiacol, vanillin, and acetovanillone, in an approximately 4:3:2 ratio, constituting ~90% of the total extracted monoaromatic content. 4-Ethanol guaiacol, vanillate, and 4-propanol guaiacol were also present. Bacterial strains that grew on minimal media supplemented with the BL extracts at 1mM total aromatic compounds included *Pseudomonas putida* KT2442, *Sphingobium* sp. SYK-6, and *Rhodococcus rhodochrous* EP4. By contrast, the extracts inhibited the growth of *Rhodococcus jostii* RHA1 and *Rhodococcus opacus* PD630, strains extensively studied for lignin valorization. Of the strains that grew on the extracts, only *R. rhodochrous* GD01 and GD02, isolated for their ability to grow on acetovanillone, depleted the major extracted monoaromatics. Genomic analyses revealed that EP4, GD01, and GD02 share an average nucleotide identity (ANI) of 98% and that GD01 and GD02 harbor a predicted three-component carboxylase not present in EP4. A representative carboxylase gene was upregulated ~100-fold during growth of GD02 on a mixture of the BL monoaromatics, consistent with the involvement of the enzyme in acetovanillone catabolism. More generally, quantitative RT-PCR indicated that GD02 catabolizes the BL compounds in a convergent manner *via* the β-ketoadipate pathway. Overall, these studies help define the catabolic capabilities of potential biocatalytic strains, describe new isolates able to catabolize the major monoaromatic components of BL, including acetovanillone, and facilitate the design of biocatalysts to valorize under-utilized components of industrial lignin streams.

## Introduction

Lignin is a heterogeneous aromatic polymer and a major component of the plant cell wall. Although it can comprise over 30% of lignocellulosic biomass, it is under-utilized in biorefineries, typically being used to power the extraction of carbohydrates (Ragauskas et al., 2014; Becker and Wittmann, 2019). Technoeconomic analyses have identified the valorization of lignin as being critical for the economic viability and sustainability of next generation biorefineries. Accordingly, considerable effort has been invested in developing processes to depolymerize lignin and convert it to value-added bioproducts (Ragauskas et al., 2014; Schutyser et al., 2018; Sun et al., 2018; Becker and Wittmann, 2019). One under-utilized, lignin-rich stream that is generated in the kraft pulping process is black liquor (BL; Johnson et al., 2019). In an effort to valorize this fraction, processes such as LignoForce™ have been developed to recover the kraft lignin (Kouisni et al., 2016). BL also contains lower molecular weight compounds, including monoaromatics, sugars, and small organic acids that represent unmined value.

The ability of phylogenetically diverse bacteria to efficiently catabolize aromatic compounds has provided an entry to developing microbial cell factories to upgrade lignin-derived aromatic compounds (LDACs) to commodity chemicals (Becker and Wittmann, 2019). Strains that have been investigated for their biocatalytic potential include *Sphingobium* sp. SYK-6 (Kamimura et al., 2017), *Pseudomonas putida* KT2440 (Weimer et al., 2020), *Novosphingobium aromaticivorans* DSM 12444 (Perez et al., 2021), *Rhodococcus opacus* PD630 (Kosa and Ragauskas, 2012), and *Rhodococcus jostii* RHA1 (Eltis and Singh, 2018). More recently, we isolated *Rhodococcus rhodochrous* EP4 for its ability to grow on alkylated guaiacols generated in the reductive catalytic fractionation (RCF) of hardwood lignin (Levy-Booth et al., 2019; Fetherolf et al., 2020). These strains not only have exceptional abilities to aerobically degrade LDACs, but also have a high natural resistance to toxic compounds (Krell et al., 2012; Xu et al., 2019). Bacteria of the genus *Rhodococcus* are of particular interest for industrial applications given their use to produce thousands of tons of acrylamide (Hughes et al., 1998).

In bacterial strains, the aerobic catabolism of aromatic compounds is normally organized in a convergent fashion, whereby “upper pathways” transform a diversity of substrates to a small number of shared intermediates, typically catechols, which are further transformed to central metabolites through “lower pathways” (Eltis and Singh, 2018; Becker and Wittmann, 2019). For example, in RHA1, upper pathways transform *p*-coumarate, ferulate, vanillate, and 4-hydroxybenzoate to protocatechuate, which is then transformed to acetyl-CoA and succinate *via* the β-ketoadipate lower pathway (Patrauchan et al., 2005; Chen et al., 2012; Otani et al., 2014). This convergent catabolism facilitates the engineering of microbial cell factories to “biologically funnel” mixtures of LDACs to commercially valuable compounds in high atom yield. For example, KT2440 has been engineered to transform alkaline pretreated lignin into muconic acid, which can in turn be hydrogenated to adipic acid, a precursor for nylon (Vardon et al., 2015). More recently, this strain was engineered to convert vanillin and vanillate from softwood BL to β-ketoadipate (Suzuki et al., 2021). Such compounds with rich functionality are more readily accessible from biomass than petroleum and can be used to develop platform chemicals and novel materials (Johnson et al., 2019).

A number of studies have investigated the potential of bacteria to transform softwood kraft lignin. In one study, Abdelaziz et al. (2019) oxidatively depolymerized lignin to a mixture of vanillin, vanillate, guaiacol, and acetovanillone. They further demonstrated that SYK-6, KT2440, and a rhodococcal strain grew on this stream. However, none of these strains completely degraded all the LDACs. Similarly, Ravi et al. (2019) used an alkaline depolymerized softwood kraft lignin as substrate for bacterial conversion, demonstrating that two *Pseudomonas* strains grew on vanillate, vanillin, and 4-hydroxybenzoate, while a rhodococcal strain degraded these compounds as well as guaiacol. In this study, acetovanillone remained as the only persistent monoaromatic. Finally, *Paenibacillus glucanolyticus* SLM1, was isolated from BL, grew on this substrate, and degraded some of the lignin to monoaromatic compounds (Mathews et al., 2014, 2016). However, the consumption of the LDACs was not analyzed.

Herein, we characterized the monoaromatic extract of softwood BL and the relative efficiency of different extraction methods. We then evaluated the ability of seven bacterial strains to grow on the BL extract and its major monoaromatic components. These included two *R. rhodochrous* strains, GD01 and GD02 that we isolated on acetovanillone and whose genomes we sequenced. Finally, we characterized growth of GD02 on the BL monoaromatic components and evaluated the involvement of predicted catabolic pathways in this growth. The results provide insights into the bacterial catabolism of LDACs present in an industrially relevant liquor stream and facilitate the design of biocatalysts for lignin valorization.

## Materials and Methods

### Chemicals and Reagents

All reagents were of analytical grade unless otherwise noted. 4-Propanol guaiacol (4PG) was synthesized and generously provided by Dr. Rui Katahira according to the method from Pepper et al. (1971).

### Characterization of BL Extracts

Black liquor was produced by Domtar Corporation (Canada) using the Lignoforce™ kraft pulping process and a mixture of white wood that included spruce, pine, and fir (Kouisni et al., 2016). Compounds were extracted from 20ml BL using 5ml of either ethyl acetate, diethyl ether, dichloromethane, or acetone. Extraction mixtures were agitated by shaker for 2h (200rpm), and were stabilized for 30min prior to phase separation.

### GC-MS Analysis

Aromatic compounds in extracts were analyzed using an Agilent Technologies (Santa Clara, U.S.A.) 6890N gas chromatograph equipped with a 30-m Agilent 190915-433 capillary column and an Agilent 5973 mass-selective detector. For monoaromatic quantification, samples were dried and derivatized using *N*,*O*-bis(trimethylsilyl)trifluoroacetamide and trimethylchlorosilane in a 50/50 mixture with pyridine. Runs were held at 90°C for 3min, and then ramped to 290°C at 12°C min^−1^ with a 10min final hold. Standard curves of each compound were run in parallel. To evaluate the substrate depletion in culture supernatant, 400μl samples were withdrawn, spiked with 5nmol of 3-chlorobenzoate as an internal standard, and extracted with an equal volume of ethyl acetate. The extracted compounds were dried under a stream of N_2_ and processed as described above. Samples were run in triplicate.

### HPLC Analysis

Organic acids in extracts were quantified using a Summit high-performance liquid chromatography (HPLC) apparatus equipped with a Shodex RI-101 detector (Dionex, United States) and an ICSep ION-300 column (300mm×7.8mm, Transgenomic, United States). The mobile phase was 0.0085N H_2_SO_4_. The column was operated at a flow rate of 0.5mlmin^−1^ at 65°C. Samples of 20μl were injected and compounds were quantified using standard curves.

For experiments on the transformation of 4PG and 4-ethanol guaiacol (4EG) by GD02, culture supernatants were analyzed using a Waters 2695 HPLC (Waters, Milford, MA, United States) equipped with a 250×4.6mm Luna® 5μm C18(2) column (Phenomenex, Torrance, CA, United States) and a UV detector. The column was operated at 0.7mlmin^−1^, and the sample was eluted using a 16.8ml linear gradient of 0.1% formic acid in H_2_O to 100% methanol. Filtered (0.2μm) samples of 100μl were injected.

### Bacterial Strains and Growth Conditions

The bacterial strains used in this study were *P. putida* KT2442, *Sphingobium* sp. SYK-6, *R. opacus* PD630, and *R. jostii* RHA1, and three strains of *R. rhodochrous*: EP4, GD01, and GD02. EP4 was recently isolated by Levy-Booth et al. (2019), and GD01 and GD02 were isolated during this study (see below). Strains were grown at 30°C on LB or M9 minimal medium supplemented with Goodies (Bauchop and Elsden, 1960) and defined organic substrates. Growth experiments were routinely performed in 250-ml shake flasks containing 50ml of culture and monitoring the optical density at 600nm (OD_600_). Growth studies in 96-well plate were performed using a Tecan Spark-Multimode Microplate Reader with shaking at 250rpm and OD_600_ recorded every 30min. Experiments were performed in triplicate.

For growth on individual substrates, substrate mixtures, and extracts, single colonies were inoculated in 5ml LB broth and grown overnight. Cells were pelleted at 1,500×*g*, washed twice with M9 media, and then used to inoculate M9-Goodies containing the substrate at an OD_600_~0.05. For growth on acetone BL extracts, solvent was evaporated under N_2_ and suspended in DMSO to prepare a 100× stock solution (100mM). Due to the turbidity of the extract, growth was evaluated by counting CFU.

For inhibition experiments, LB broth was amended with BL extract to a total concentration of 1mM monoaromatic compounds and inoculated to an OD_600_~0.05 with cells grown overnight in the same media. LB without BL extract served as a control.

### Enrichment and Isolation

Enrichment cultures were inoculated with compost from The University of British Columbia farm, contained 1mM acetovanillone (≥98% Sigma-Aldrich, St. Louis. United States) as sole carbon source in M9 with Goodies, and were incubated with shaking at 200rpm at either 30 or 37°C. The cultures were serially transferred three times, with acetovanillone increased to 2mM for the final two passages. Isolates were then obtained by streaking cultures on homologous media with 1mM acetovanillone and 1.5% agar. Individual colonies were streaked three times successively to obtain pure isolates. Isolates were tested for growth on 1mM acetovanillone in liquid medium, and the removal of acetovanillone was measured by gas chromatography–mass spectrometry (GC-MS). Only two of nine isolates grew on acetovanillone, GD01, isolated at 37°C, and GD02, isolated at 30°C.

### Transformation of 4PG and 4EG by GD02 Cells

Cells of GD02 were grown overnight in 5ml LB and washed as described in “Bacterial Strains and Growth Conditions.” These cells were used to inoculate M9-Goodies supplemented with 1mM mixture of monoaromatics in the same proportion as in the BL acetone extract. The medium was inoculated to an OD_600_~0.05 and cells were harvested when the culture reached mid-log phase. The harvested cells were concentrated 25× and incubated with each of 10mM 4PG and 4EG at 30°C. Supernatant samples were withdrawn at different times, acidified with acetic acid, and analyzed using HPLC.

### Genome Sequencing and Bioinformatics

Genomic DNA was extracted from strains GD01 and GD02 using FastDNA SPIN kit for soil (MPBio, Solon, OH, United States). Illumina sequencing was performed by the Microbial Genome Sequencing Center (MiGS, Pittsburgh, PA, United States) on the NextSeq2000 platform, and generated 3.3 million 150-bp pair-end reads with 111-fold sequencing coverage. *De novo* draft genomes were assembled by first processing and decontaminating reads with BBMap (Bushnell et al., 2017). Quality filtering and trimming of reads used BBDuk (Bushnell et al., 2017). Cleaned and filtered reads were then assembled using SPAdes 3.13.0 (Bankevich et al., 2012), MeDuSa 1.6 scaffolding (Bosi et al., 2015), and gap filling used SSPACE (Boetzer et al., 2011). Gene annotation used DIAMOND (Buchfink et al., 2015) and BLASTp against the Protein Data Bank (threshold of *E*-value 10^−3^). Average nucleotide identity (ANI) was calculated using FastANI (Jain et al., 2018).

### RNA Extraction and RT-qPCR

Cultures for quantitative reverse transcription PCR (RT-qPCR) were grown on M9-Goodies supplemented with 1mM mixture of monoaromatics in the same proportion as in the BL acetone extract or 1mM citrate and harvested after 7h (OD_600_ 0.13) and 11h (OD_600_ 0.25). Cells were flash frozen and stored at −80°C for later RNA extractions. Total RNA was extracted using Trizol and cleaned up by PureLink RNA mini kit (Thermo Fisher Scientific). Cells were suspended in Trizol and disrupted using a bead beater (MP Biomedicals FastPrep-24 Solon, OH) with six rounds of 30s with speed set at 5.5ms^−1^, and 5min pauses between rounds. RNA samples were further treated with Turbo DNase I (Thermo Fisher Scientific) and the DNase Inactivation Reagent. RNA was reverse transcribed to cDNA using SuperScript™ VILO™ cDNA Synthesis Kit (Thermo Fisher Scientific). Quantitative PCR was performed using a StepOne Plus Real-Time PCR System (Thermo Fisher Scientific) using the PrimeTime Gene Expression master mix (Integrated DNA Technologies). Primers and probes are listed in Supplementary Table 1. The cycling conditions were: 95°C for 3min, then 40cycles of: 95°C for 5s and 60°C for 30s. Standard curves for all genes were made using 10× serial dilutions of synthesized target DNA gblocks from (0.1ng–0.1fg). Transcript levels were normalized to the GD02 *sigA* cDNA and compared to expression in GD02 cells growing on 1mM citrate.

## Results

### Identification of Monoaromatic Compounds in BL

To identify the aromatic compounds present in BL and to evaluate the efficiency of their isolation, we extracted softwood BL that had been subjected to the Lignoforce™ process with each of four solvents: ethyl acetate, diethyl ether, dichloromethane, and acetone. As described in Supplementary Material, the Lignoforce™ process involves first oxidizing the BL, then neutralizing it with CO_2_ and then rinsing with sulfuric acid to yield the BL filtrate for liquid extraction (Kouisni et al., 2016). Using a single extraction with 1:4 (v/v) solvent:BL, the greatest total amount of monoaromatic compounds was obtained using acetone while diethyl ether yielded the least (Table 1). In the acetone extract, guaiacol, vanillin, and acetovanillone were the most abundant monoaromatic compounds, collectively constituting ~90% of the total monoaromatic content in an approximately 4:3:2 molar ratio. 4EG, 4PG, catechol, and vanillate were also detected. Comparison with the BL indicated that acetone extracted 35% of the neutral aromatic compounds from the BL and 3% of the vanillic acid. The acetone extract also contained significant quantities of formate and acetate (Table 1). By comparison, diethyl ether extracted around half the neutral aromatic compounds that acetone did.

**Table 1:** Components of Lignoforce™ industrial black liquor (BL) filtrate from mixed softwood species^a^.

	Extraction solvent			
Parameter/component	Acetone	Dichloromethane	Diethyl ether	Ethyl acetate
δD, δP, δH^b^	15.5, 10.4, 7	17, 7.3, 7.1	14.5, 2.9, 4.6	15.8, 5.3, 7.2
Total solids (wt%)	2.8	0.5	0.5	0.5
Guaiacol^c^	7.4	8.8	6.6	8.0
Vanillin	5.5	2.9	1.1	4.3
Acetovanillone	3.5	3.4	1.5	3.6
4-propanol guaiacol	0.6	0.4	0.3	0.5
4-ethanol guaiacol	0.4	0.2	0.1	0.3
Catechol	0.9	ND^d^	0.3	0.6
Vanillic acid	0.5	ND	ND	ND
Total phenolics (mM)^e^	18.8	15.7	9.9	17.3
Extraction efficiency (%)^f^	34.9	29.9	18.9	33.0
Formic acid	101	17	22	34
Acetic acid	38	ND	ND	ND
Succinic acid	0.8	ND	ND	ND
Total acids (mM)^e^	140	17	22	34

a Spruce, pine, and fir.

b Hansen solubility parameters: dispersion, polar, and hydrogen bonding components of cohesive energy density of the solvent (Hansen, 2007).

c Concentration of compounds in extracts provided in mM based on triplicate extractions. Errors did not exceed 5%.

d ND, not detected.

e Trace amounts (~0.1mM) of lactic acid, 2-hydroxybutyric acid, 4-hydroxy-2-pentenoic acid, 4-hydroxy-3-pentenoic acid, and 2,5-dimethoxybenzoic acid were also detected in the acetone extract, but not in any of the others.

f Estimated for neutral monoaromatics.

### Isolation of *R. rhodochrous* GD01 and GD02

**Figure 1:**
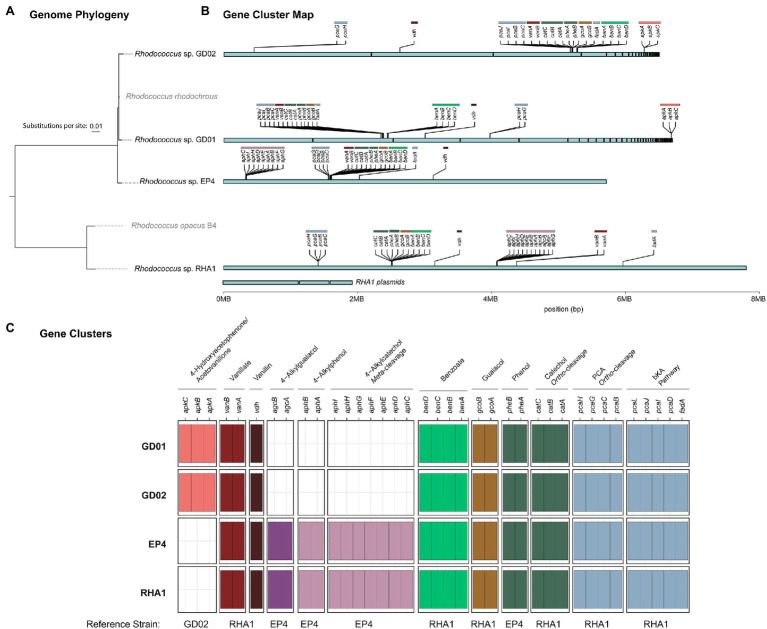
Presence of select aromatic catabolic genes in the GD01, GD02, EP4, and RHA1 genomes. **(A)** Genome phylogeny created using IQ-Tree2 from MAFFT alignment of 120 concatenated single-copy genes showing the placement of GD01, GD02, and EP4 in the *Rhodococcus rhodochrous* clade. **(B)** The assembly contigs for each genome are plotted to show the location of gene clusters involved in degradation of select aromatic compounds. **(C)** Summary plot of select aromatic catabolic genes in the GD01, GD02, and EP4 (accession number GCA_003004765.2) and RHA1 (accession number GCA_000014565.1) genomes. A custom database of genes for each pathway was assembled, and the presence/absence of genes in each strain were identified by BLAST search, with a threshold of 25% identity and *E*-value of 1E^−30^.

The identification of acetovanillone in BL extracts prompted us to isolate strains able to grow on this compound. Enrichment cultures were inoculated with compost and had acetovanillone as the sole organic substrate. After serial transfers of the enrichment cultures and streaking on homologous solid medium, two isolates that grew on acetovanillone were obtained and identified as GD01 and GD02. Sequencing the genomes of the strains yielded assemblies of 6.35 and 6.29Mb for GD01 and GD02, respectively, that were estimated to be 99.2% complete (Supplementary Table 2). GD01 and GD02 were classified as *R. rhodochrous* strains (Figure 1) based on the nucleotide sequence of their 16S rRNA genes, ANI, and phylogenetic placement (Chaumeil et al., 2019). The 16S rRNA genes (27F-1492R; Lane, 1991) of GD01 and GD02 shared 100% sequence identity with that of *R. rhodochrous* EP4 (Levy-Booth et al., 2019). More generally, the GD01 and GD02 genomes were very closely related to each other, with an ANI of 99.1%, as well as to 5.72Mb genome of EP4 (ANI 97.9%).

**Figure 2:**
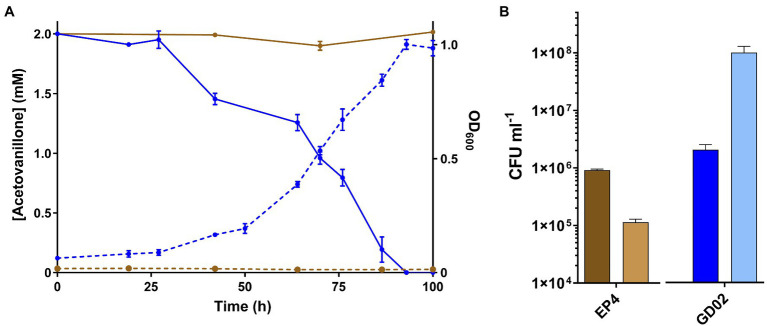
Growth of GD02 on acetovanillone. **(A)** GD02 was grown in M9 minimal medium with 2mM acetovanillone at 30°C. Solid lines represent concentration of acetovanillone as determined using GC-MS, and dashed lines represent OD_600_. Blue and brown lines represent data for GD02 and EP4, respectively. **(B)** CFU ml^−1^ at 0h (dark) and 100h (light). Data points show average of triplicate experiments and the vertical bars the SD.

GD02 grew on 2mM acetovanillone to stationary phase within 100h in shake flasks (Figure 2A). Growth on acetovanillone was verified by plating CFUs (Figure 2B). GC-MS analysis indicated that acetovanillone was completely removed from the medium during growth (Figure 2A), with no metabolites detected. EP4 did not grow on acetovanillone.

### Five Bacterial Strains Grew on BL Extracts

We assessed the toxicity of the BL extracts against a panel of seven bacterial strains known to grow on one or more of the major aromatic components of the BL: *Sphingobium* sp. SYK-6 (Kamimura et al., 2017), *P. putida* KT2442 (Weimer et al., 2020), *R. jostii* RHA1 (Eltis and Singh, 2018), *R. opacus* PD630 (Kosa and Ragauskas, 2012), *R. rhodochrous* EP4 (Fetherolf et al., 2020), *R. rhodochrous* GD01, and *R. rhodochrous* GD02. These strains were phylogenetically diverse, with the exception of the EP4, GD01, and GD02, which were included due to their distinct growth phenotypes.

For the toxicity experiments, solvents were evaporated from the different extracts and the residual materials were suspended in DMSO. Extracts were added to LB media such that the final concentration of BL aromatic compounds was 1mM (final 1% DMSO). Among the seven tested strains, RHA1 and PD630 were the only ones to be completely inhibited by all four extracts (Supplementary Figure 1). At the other end of the spectrum, KT2442 and SYK-6 were the only strains whose growth on LB was not inhibited by any of the four extracts. The growth of EP4, GD01, and GD02 was delayed by the addition of acetone extract (i.e., longer lag phase), and was inhibited by the other three extracts. Nevertheless, the addition of the acetone extract increased the growth yield of the three *R. rhodochrous* strains, consistent with assimilation of BL-derived compounds. The dichloromethane and diethyl ether extracts were the most toxic, completely inhibiting the growth of the five *Rhodococcus* strains. Overall, these results indicate that the acetone-extracted BL is the least toxic of the extracts. Consequently, it was selected for further experiments as a growth substrate.

**Figure 3:**
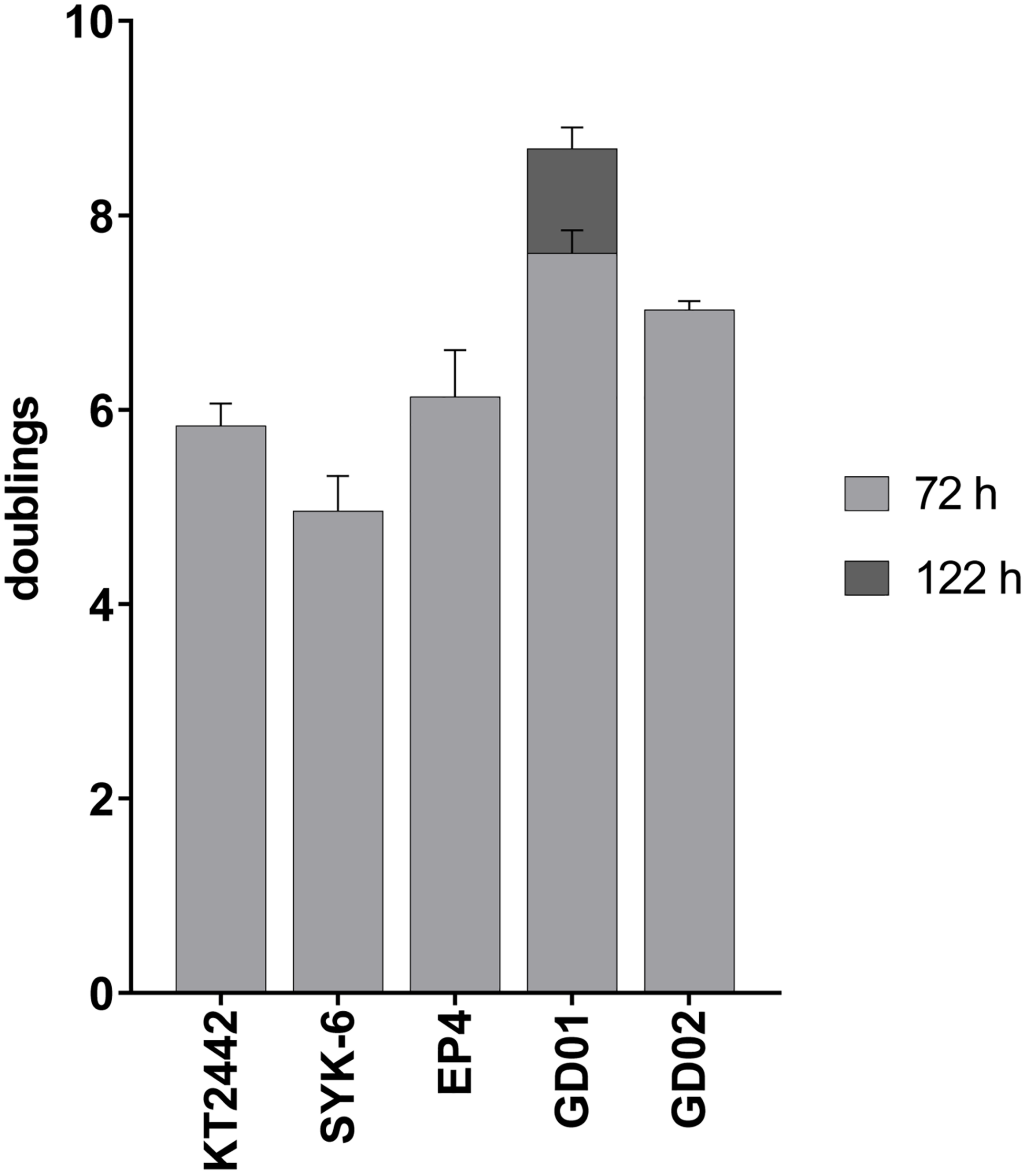
Growth on black liquor extract. Strains were grown at 30°C on M9 minimal medium supplemented with acetone-extracted monoaromatic compounds to a total concentration of 1mM. All strains hit stationary phase after 72h, except for GD01 which was monitored until 122h. Experiments were performed in triplicate.

To study strains able to catabolize monoaromatics present in BL, we first assessed the growth of KT2442, SYK-6, EP4, GD01, and GD02 on BL acetone extracts such that the final concentration of monoaromatics was 1mM. All strains showed robust growth, undergoing at least five doublings (Figure 3). Although GD01 underwent the greatest number of doublings, it was the only strain that did not reach stationary phase within 72h. A similar experiment was performed in a plate reader using a mixture of the aromatic compounds in the same proportion as in the BL acetone extract to a total concentration of 2mM as sole growth substrate in minimal medium (Supplementary Figure 2). Consistent with the growth on BL extracts, GD01 and GD02 grew to the highest OD on this mixture. The relatively low yield of KT2442 on the compound mixture vs. on the acetone extract could be due to the presence of additional substrates in the extracts, such as formate and acetate (Table 1).

### Depletion of Monoaromatic Compounds From BL Extracts

**Figure 4:**
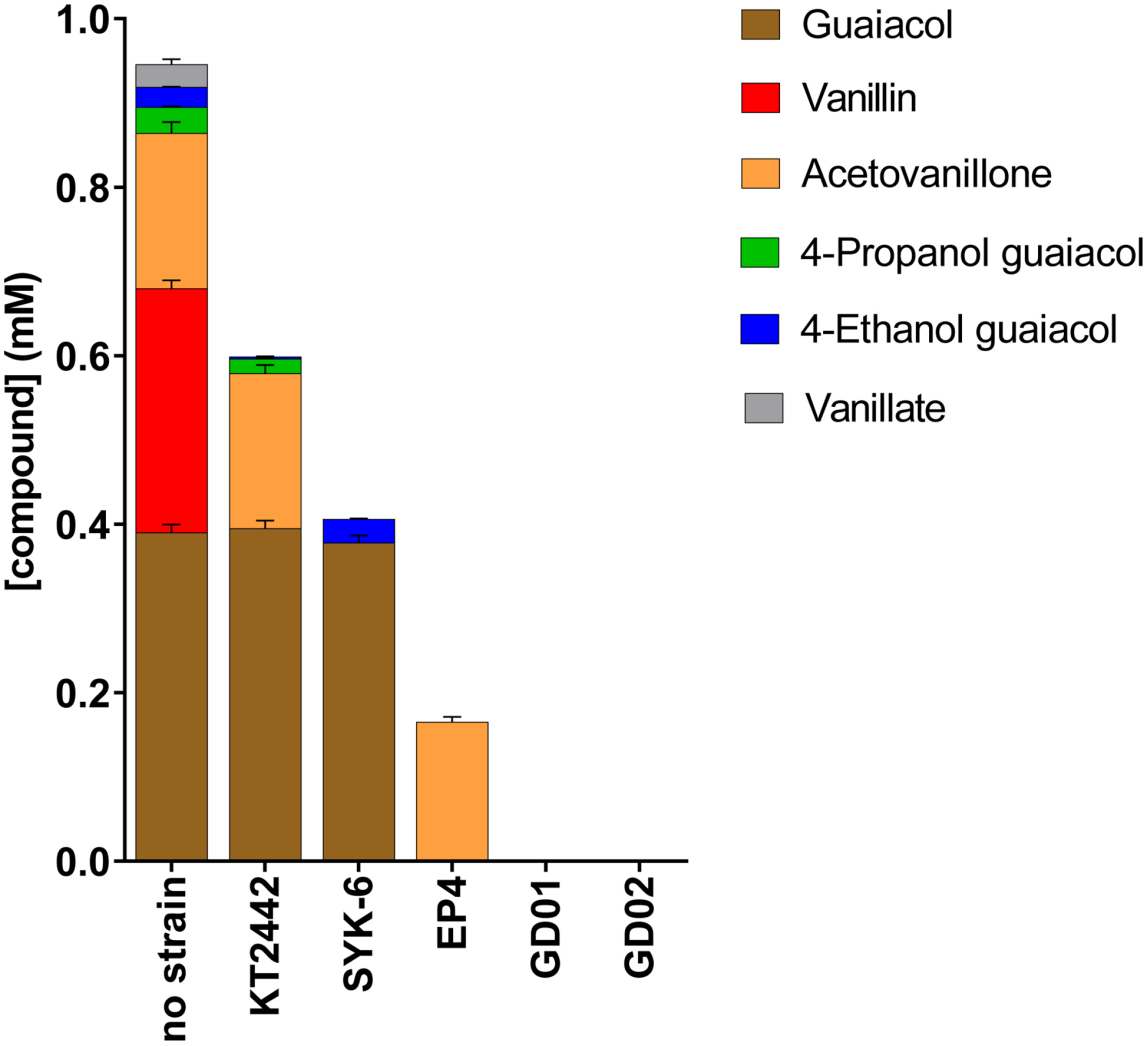
Depletion of black liquor aromatic compounds by bacterial strains. Strains were incubated for 72h on minimal medium amended with BL extract for a final concentration of 1mM total monoaromatic compounds. Compounds were quantified using GC-MS.

To identify the aromatic compounds in the BL extract depleted by each strain, we analyzed spent culture supernatants using GC-MS. Catechol was not detected in the no-strain control, presumably due to its non-enzymatic oxidation. As summarized in Figure 4, only GD01 and GD02 depleted the six major monoaromatic compounds in the acetone extract after 72h. EP4 depleted all the compounds except acetovanillone. Among the non-rhodococcal strains, KT2442 completely depleted the vanillin and vanillate, and some of the 4EG. SYK-6 did not detectably deplete the guaiacol or 4EG, but completely depleted the other monomers, including acetovanillone. However, the SYK-6 did not grow on acetovanillone alone. Moreover, in monitoring the depletion of acetovanillone in the presence of each of the other monoaromatics, we found that this depletion occurred in the presence of vanillate. Consistent with vanillin being catabolized *via* vanillate, the addition of vanillin also induced acetovanillone depletion (Supplementary Figure 3). The results on the depletion of monoaromatics from BL extracts were replicated using the mixture of these compounds in defined medium. Finally, the total amount of compounds consumed correlated with the biomass yield of each strain growing in the mixture (Supplementary Figure 2). Thus, GD01 and GD02, the only two strains that depleted all six compounds, yielded the most biomass on the mixture. Because GD02 grew faster and more reproducibly in flasks than did GD01, it was selected for further studies.

### Identification of Pathways Responsible for the Catabolism of BL Aromatic Compounds

To identify the pathways potentially responsible for the catabolism of monoaromatic compounds in BL, we searched the draft genome sequences of GD01 and GD02 for aromatic catabolic genes using the RHA1 and the EP4 genomes as references. Although the EP4 genome is more similar to that of GD02, many of the aromatic catabolic genes have been validated in RHA1. Several gene clusters and individual genes were identified that encode catabolic pathways for the compounds degraded by GD02 (Figure 1). These include genes encoding guaiacol *O*-demethylase (*gcoAB*), vanillin dehydrogenase (*vdh*), vanillate *O*-demethylase (*vanAB*), and a convergent β-ketoadipate pathway for the catabolism of catechol and protocatechuate to central metabolites (Supplementary Table 3). Consistent with these predictions, GD02 grew on each of 2mM vanillate, guaiacol, and vanillin (Supplementary Figure 4), with approximate doubling times of 3.3h for the first two substrates and 16h for vanillin at 30°C. As with acetovanillone, growth on vanillin was preceded by a prolonged lag phase.

A striking difference between the GD02 and EP4 genomes despite their high ANI was the absence of *agc* and *aph* genes in GD02 (Figure 1). These genes are responsible for the catabolism of 4-alkylguaiacols and 4-alkylphenols in EP4 (Levy-Booth et al., 2019; Fetherolf et al., 2020). Consistent with this finding, GD02 did not grow on either 4-propylguaiacol or 4-ethylphenol. Indeed, the GD02 genome does not appear to contain any genes encoding extradiol dioxygenases, suggesting that most, if not all, of the aromatic compounds catabolized by this bacterium are funneled through the β-ketoadipate pathway, which contains intradiol dioxygenases.

**Figure 5:**
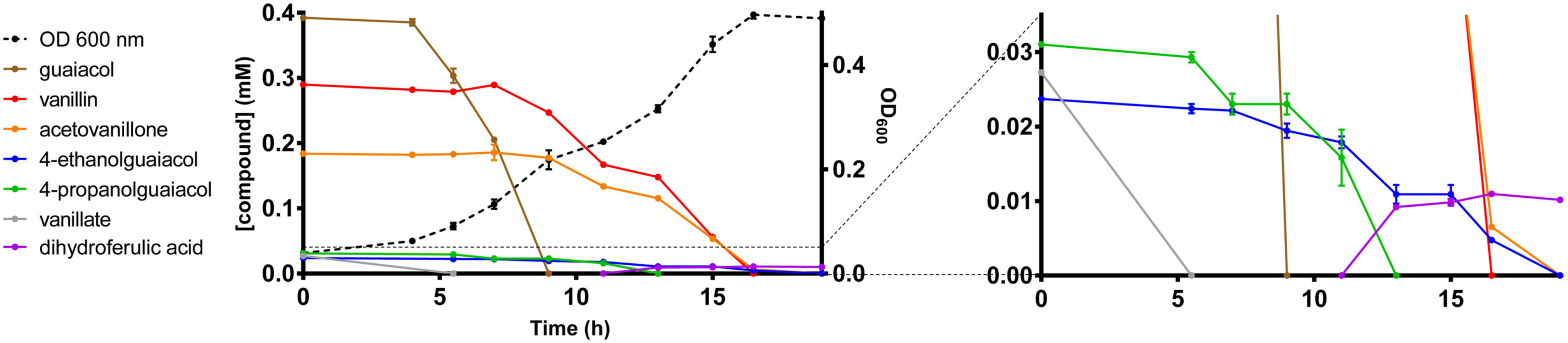
Growth of GD02 on a mixture of black liquor aromatic compounds. GD02 was grown at 30°C in defined medium with a mixture of aromatic compounds in the same proportions as in the BL acetone extract, total concentration 1mM. Compounds were measured in the culture supernatant using GC-MS analysis. Data points represent the average of triplicate experiments and the error bars the SD. The panel on the right depicts the compounds present at lower concentrations.

To date, there have been no reports describing the catabolism of acetovanillone. However, the catabolism of the chemically related compound, 4-hydroxyacetophenone, has been described in *Aromatoleum aromaticum* strain EbN1 (Wohlbrand et al., 2008). This catabolism is initiated by a three-component, biotin-dependent carboxylase, XccBCA, comprising a biotin carboxyl carrier protein (BCCP), a biotin carboxylase (BC), and a carboxyl transferase (CT), respectively. Interestingly, the GD01 and GD02 draft genomes contain genes encoding a homolog of this enzyme, whose subunits share 45–50% amino acid sequence identity with the EbN1 homologs (Table 2). These genes do not occur in EP4 (Figure 1) which does not grow on acetovanillone (Figure 2A). Based on these observations, we propose that the genes, annotated as *apkCBA*, encode a carboxylase that is involved in the catabolism of acetovanillone, an alkyl-phenyl ketone, in GD02 and, by extension, GD01. To further understand the catabolism of the monoaromatic compounds by GD02, we monitored their consumption during growth on a mixture of the compounds. Consistent with the growth of GD02 on the individual compounds, guaiacol and vanillate were the first compounds to be consumed (Figure 5). Similarly, vanillin and acetovanillone were consumed after a significant lag phase. 4EG and 4PG were depleted at about the same time as the vanillin was consumed. Interestingly, dihydroferulate was detected in the culture supernatant at this time, suggesting that it was produced by the enzymatic oxidation of 4PG. By contrast, the presumed oxidation product of 4EG, homovanillate, was not detected. Importantly, GD02 did not grow on either 4EG or 4PG. To investigate the ability of GD02 to transform 4PG and 4EG, cells grown on the mixture of monoaromatics were incubated with each of the alkylguaiacols. As summarized in Supplementary Figure 5, the cells completely transformed 4PG into two compounds, one of each was dihydroferulate, but only partially transformed 4EG to homovanillate.

**Table 2:** Annotation of GD02 gene targets.

Gene	Description	Gene ID^a^	Ref strain	Accession no^b^	%ID^c^	References
*catA*	Catechol 1,2-dioxygenase	3_1052	RHA1	WP_009475036.1	69.0%	Patrauchan et al., 2005
*pcaH*	Protocatechuate 3,4-dioxygenase, β subunit	1_410	RHA1	WP_009474041.1	48.7%	Patrauchan et al., 2005
*vanA* ^d^	Vanillate *O*-demethylase, oxygenase	3_1042	RHA1	WP_011596645.1	30.1%	Chen et al., 2012
*vdh*	Vanillin dehydrogenase	2_1324	RHA1	WP_011595659.1	52.4%	Chen et al., 2012
*gcoA*	Guaiacol *O*-demethylase, cytochrome P450	3_1059	RHA1	WP_011595125.1	76.4%	Fetherolf et al., 2020
*apkC*	Putative alkyl-phenyl ketone carboxylase, γ subunit	13_4	*Aromatoleum aromaticum* EbN1	CAI06288.1	46.5%	Wohlbrand et al., 2008
*apkB*	Putative alkyl-phenyl ketone carboxylase, β subunit	13_5	*Aromatoleum aromaticum* EbN1	CAI06287.1	50.2%	Wohlbrand et al., 2008
*apkA*	Putative alkyl-phenyl ketone carboxylase, α subunit	13_6	*Aromatoleum aromaticum* EbN1	CAI06286.1	44.8%	Wohlbrand et al., 2008

a Gene ID in GD02 assembly.

b Indicated genes were reciprocal best hits in the reference strain.

c Amino acid sequence identity. In addition, *CatA*, *PcaH*, *VanA*, *Vdh*, and *GcoA* were 100% identical in amino acid sequence to the corresponding gene products in EP4 (Levy-Booth et al., 2019).

d The coding sequence in the assembly is 42% complete. Importantly, vanB is immediately downstream of vanA.

**Figure 6:**
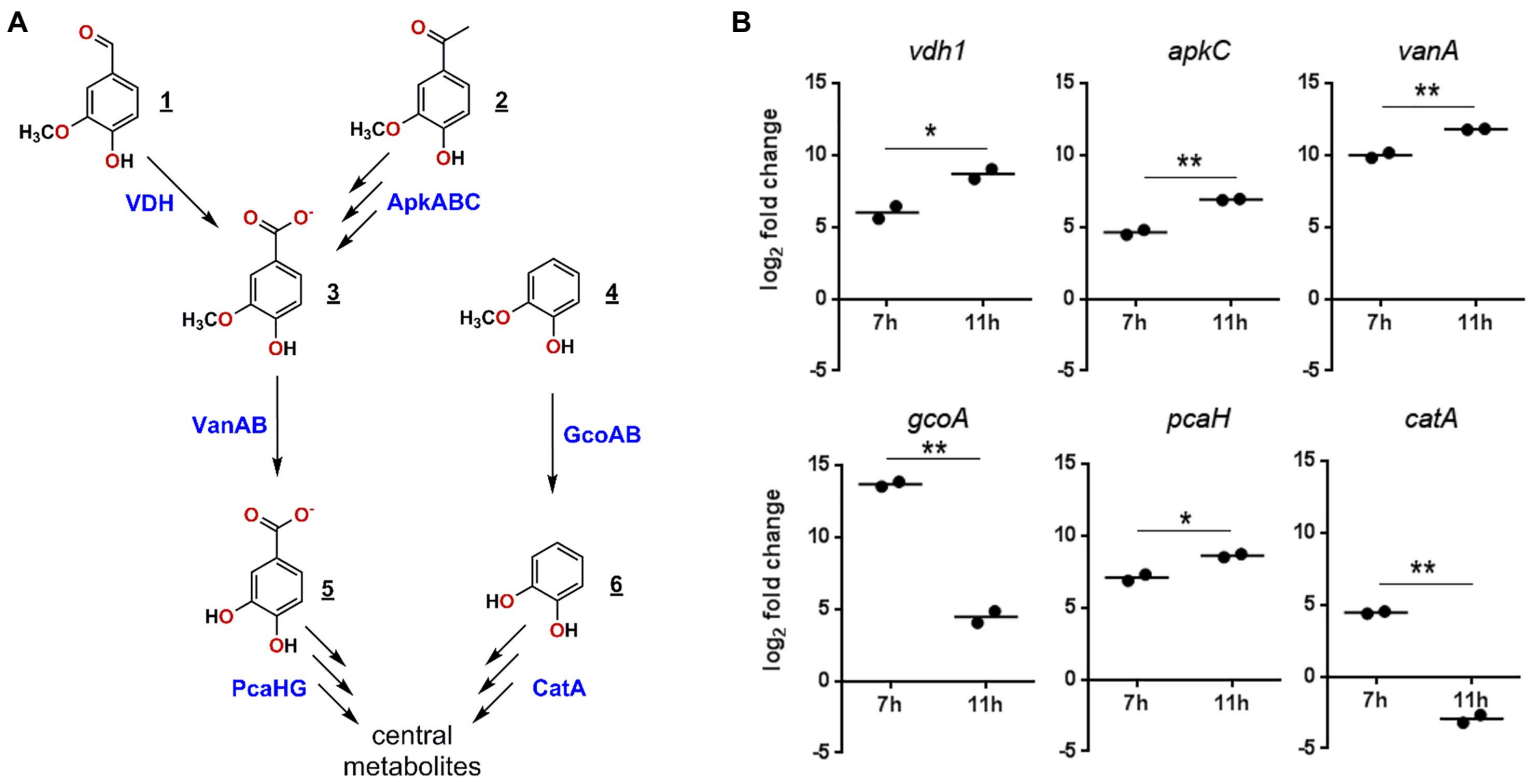
Expression of selected genes in GD02 during growth on a mixture of black liquor compounds. **(A)** Enzymes predicted to be involved in the catabolism of the major BL monoaromatic compounds and whose genes were investigated using RT-qPCR. Compounds are: 1, vanillin; 2, acetovanillone; 3, vanillate; 4, guaiacol; 5, protocatechuate; and 6, catechol. **(B)** Expression RT-qPCR of key catabolic genes in GD02 after 7 and 11h of growth on 1mM of a mixture of monoaromatic compounds in the same proportion as in the BL extracts. Expression was evaluated using RT-qPCR and are reported as log_2_ expression vs. cells growing on 1mM citrate after normalization to *sigA* expression. Values are the average of two technical replicates. Significance was determined by *t*-test, where *p* values are: ^*^<0.05, ^**^<0.01.

We next evaluated the functionality of the predicted GD02 pathways by using RT-qPCR to assess the expression of select genes (Figure 6A; Table 2) during growth of the bacterium on BL extract. Based on the profile of compound consumption (Figure 5), we sampled RNA of cultures of GD02 growing on a mixture of the BL aromatics at 7 and 11h. Gene expression was compared to that in GD02 growing on 1mM citrate. As shown in Figure 6B, vanillin (*vdh*), guaiacol (*gcoA*), vanillate (*vanA*), and acetovanillone (*apkC*) catabolic genes were upregulated at least 30-fold during growth on a mixture of BL monoaromatics vs. citrate. Consistent with the substrate depletion profile (Figure 5), *gcoA* transcripts were~100-fold more abundant at 7 vs. 11h, while *vdh* and *apkC* transcripts were more abundant at 11h. Finally, the two tested lower pathway genes were also upregulated during growth on BL extract. Like *gcoA*, *catA* transcripts were more abundant at the earlier sampling time, consistent with the prediction that guaiacol is the only compound catabolized *via* catechol. The expression profile of *pcaH* is consistent with the catabolism of vanillin and acetovanillone *via* the protocatechuate branch of the *ortho*-cleavage pathway.

## Discussion

Guaiacol, vanillin, and acetovanillone are the most abundant monoaromatic compounds in Lignoforce™ softwood BL. These are the same major monoaromatic compounds that have been reported in other depolymerized softwood lignin samples, including an oxidative depolymerized Lignoboost softwood kraft lignin (Abdelaziz et al., 2019), an alkaline depolymerized pine kraft lignin (Indulin AT; Prothmann et al., 2017; Ravi et al., 2019), and OCF pine lignin (Zhu et al., 2020). This suggests that these compounds are characteristic monoaromatic compounds generated by oxidative depolymerization of softwood lignin. 4PG and 4EG constituted a relatively minor proportion of the BL monoaromatic compounds reported here (~5% total), but have not been reported in similar softwood lignin samples. This may reflect differences in the fractionation methods or the origin of the lignin. Interestingly, 4PG is one of the major components of RCF of *Eucalyptus* (Chen et al., 2020) and corn stover (Fetherolf et al., 2020).

The recovery of monoaromatics and small organic acids, as well as the toxicity of the BL extracts depended on the solvent used for the extraction. Thus, increasing the polarity of the solvents, as reflected by the Hansen solubility parameters (Table 1), improved the yield of monoaromatics and small organic acids while at the same time diminishing the toxicity of the extracts. The improved recovery of the monoaromatics and acids using higher polarity solvent is consistent with previous reports (Jipa et al., 2009; Yang et al., 2021). However, there are no studies referencing the toxicity of the extracted compounds to bacteria. The strains examined in this study fall into two phyla, Proteobacteria and Actinobacteria, and have been previously characterized for their abilities to metabolize LDACs (Kosa and Ragauskas, 2012; Salvachúa et al., 2015; Kamimura et al., 2017; Levy-Booth et al., 2019; Ravi et al., 2019). Acetone extracts contained the highest amount of monoaromatics and were the least toxic to bacteria, suggesting that the inhibition of growth was not due to the monoaromatics. The two Proteobacteria, KT2442 and SYK-6, stood out as the only strains not inhibited by any of the four extracts. Interestingly, depolymerized softwood LignoBoost lignin inhibited the growth of *R. opacus* DSM 1069 but not of KT2440 and SYK-6 (Abdelaziz et al., 2019), in agreement with this study.

The ability of the different bacterial strains to grow on the BL extract, replicated using a mixture of these compounds, is largely consistent with the known or predicted catabolic capabilities of the respective bacteria. For example, vanillin and vanillate were consumed by all five strains, in agreement with the presence of *vdh* and *vanAB* genes in their genomes. Similarly, the three *Rhodococcus* strains depleted guaiacol, consistent with them encoding GcoAB, a cytochrome P450 system responsible for the *O*-demethylation of guaiacol to catechol (Fetherolf et al., 2020). The inability of KT2442 and SYK-6 to assimilate guaiacol in a mixture of monoaromatics was also observed in previous reports (Abdelaziz et al., 2019; Ravi et al., 2019). GD01 and GD02 were the only strains that consumed all the identified monoaromatics, which is consistent with these strains reaching the highest OD_600_ on a mixture of the monoaromatic compounds. SYK-6 was also able to deplete acetovanillone from BL; consistent with a previous report that SYK-6 depletes acetovanillone when growing on a mixture of compounds (Abdelaziz et al., 2019). As demonstrated here, that depletion depends on the presence of vanillate. Further studies are needed to determine the extent and mechanism of this depletion. Although 4PG and 4EG were minor components of BL extracts, it is nevertheless interesting that SYK-6 degraded 4PG but not 4EG. It is possible that the side chain of 4PG is oxidized to a carboxylate and further metabolized by the same pathway as ferulate (Masai et al., 2002). 4EG, with its shorter side chain, would not be degraded by this pathway. Further studies are required to test this hypothesis.

GD01 and GD02 were the only strains that grew on all the major monoaromatic compounds in the BL, and are the only strains reported to grow on acetovanillone. These findings are consistent with previous reports by Abdelaziz et al. (2019) and Ravi et al. (2019) in which known bacterial strains were able to grow on depolymerized softwood kraft lignin although none depleted all the monoaromatics. The comparative genomic data, bioinformatic data, and the RT-qPCR analysis strongly suggest that acetovanillone catabolism involves the carboxylation of the acetovanillone moiety, similar to 4-hydroxyacetophenone catabolism in EbN1 (Wohlbrand et al., 2008). Thus, GD01 and GD02 consumed acetovanillone, but a very closely related strain, EP4, did not. Notably, the *apk* genes occur in GD01 and GD02, but not EP4. Consistent with the involvement of the carboxylase in acetovanillone catabolism, *apkC* was upregulated during growth on BL extract. While it is unclear how acetovanillone carboxylation contributes to its catabolism, we note that this reaction would yield a β-keto acid similar to that generated in the catabolism of ferulate by RHA1, which yields vanillate and acetyl-CoA (Otani et al., 2014). Efforts to elucidate acetovanillone catabolism in GD01 and GD02 are ongoing.

The genomic and RT-qPCR analyses further indicate that in GD02, all the major BL LDACs are funneled through the β-ketoadipate pathway (Figure 6). More specifically, guaiacol is catabolized *via* the catechol branch of the pathway while vanillin and potentially acetovanillone are catabolized by the protocatechuate branch. Importantly, the *gcoA* and *vanA* transcripts were most highly abundant at the earlier sampling time, consistent with guaiacol and vanillate being the first compounds in the mixture to be depleted. Similarly, the higher abundance of *vdh* and *apkC* during the second sampling time is consistent with the delayed catabolism of vanillin and acetovanillone. The upregulation of *vanA* and *pcaH* at the later sampling time is further evidence that vanillin and acetovanillone are catabolized *via* vanillate and the β-ketoadipate pathway. Overall, the catabolism of BL LDACs by GD02 provides a striking example of convergence, an organizational principle of the catabolism of aromatic compounds documented in other bacteria, including rhodococci (Eltis and Singh, 2018).

Overall, our results establish that bacteria are able to catabolize the major monoaromatic components of Lignoforce™ softwood BL. Acetone appears to be the best extraction solvent, recovering the highest quantities of monomers from this liquor stream, and the lowest quantities of growth-inhibiting compounds. Of the five strains that grew on the BL extracts, only GD01 and GD02 catabolized all the identified aromatic monomers. The genes responsible for catabolizing the various LDACs in GD02 were provisionally identified providing a basis for designing biocatalysts to valorize BL and other under-utilized lignin-rich streams. The ability of GD02 to grow on acetovanillone is of particular interest. More specifically, elucidating the acetovanillone catabolic pathway is critical to exploiting its biocatalytic potential and designing microbial cell factories to valorize lignin streams.

## Data Availability Statement

The GD01 and GD02 genome assemblies can be downloaded from NCBI at accessions JAHSQN000000000 and JAHRXG000000000, respectively.

## Author Contributions

LN performed genomic and transcriptomic analysis and growth experiments, and co-wrote the manuscript. GD isolated strains GD01 and GD02, performed genome sequencing and annotation experiments, and helped to write the manuscript. DL-B assisted with genome assembly, annotation, and analyses. JL performed the RT-qPCR study. MC and S-KJ prepared and analyzed black liquor extracts. SM and SR designed extraction studies and edited the manuscript. WM helped to design the study and edited the manuscript. LE co-designed the study, helped interpret results, and co-wrote the manuscript. All authors contributed to the article and approved the submitted version.

## Funding

This study was supported by a research contract from Genome BC (GEN005) and the BC BioProducts Alliance. LE is the recipient of a Canada Research Chair.

## Conflict of Interest

The authors declare that the research was conducted in the absence of any commercial or financial relationships that could be construed as a potential conflict of interest.

## Publisher’s Note

All claims expressed in this article are solely those of the authors and do not necessarily represent those of their affiliated organizations, or those of the publisher, the editors and the reviewers. Any product that may be evaluated in this article, or claim that may be made by its manufacturer, is not guaranteed or endorsed by the publisher.
