# Supplementary material for: Bacterial Transformation of Aromatic Monomers in Softwood Black Liquor
Source: Front Microbiol. 2021 Sep 10;12:735000. doi: 10.3389/fmicb.2021.735000 (PMC8461187; doi:10.3389/fmicb.2021.735000)
Supplement: Supplementary file 1 [file Data_Sheet_1.PDF]

Supplementary Material

Bacterial transformation of aromatic monomers in softwood black liquor

Navas *et al*.

1 Supplementary Methods

1.1 Black liquor preparation

Black liquor (BL) was recovered from an industrial operation for the production of chemical sulfate pulp by Domtar Corporation at their Kamloops, British Columbia facility. The operation used a continuous digester with a mixed softwood species that generated a pulp with a Kappa number of 28.7. The recovered and concentrated BL had a solids content of 41.03% with a pH of 13.7. The BL underwent a pilot-scale oxidation process by FPInnovations to reduce the sulfide concentration via their patented Lignoforce™ process. The oxidized BL was subsequently acidified with CO_2 and the resulting precipitated lignin was separated from liquor using a filter press. The filtrate was then recovered, analyzed by HPLC and extracted for its monoaromatics.

1.2 Black liquor monoaromatics quantification

Aromatic compounds in BL samples were analyzed using a Waters 2695 HPLC (Waters, Milford, MA) equipped with a 250 × 4.6 mm Luna® 5 µm C18(2) column (Phenomenex, Torrance, CA) and a UV detector. Samples were acidified to 10% acetic acid and centrifuged for 5 min at maximum velocity. Filtered (0.2 µm) samples of 50 µL were injected. The column was operated at 0.7 mL min^{-1} and the sample was eluted using a 16.8 mL linear gradient of 0.1% formic acid in H_2O to 100% methanol. Standard curves of monoaromatic compounds were run in parallel.

2 Supplementary Tables

Supplementary Table 1: Primers and probes used for RT-qPCR experiments

Gene	Forward Primer	Reverse Primer	Probe
catA	TGC GAC CGA CAA GTT CAA	TCG GCG ATC TAC CAG GAC GTG ATC	/56-FAM/TC GGC GAT C/Zen/T ACC AGG ACG TGA TC/3IABkFQ/
pcaH	CAC AAG AAG GAC ACC TAC CTC	GAT CGT CCG GAA CTG GTA AG	/56-FAM/CC ATC GAT C/Zen/C GAA CTT CGG TGG TT/3IABkFQ/
vanA	GGG CGA GAT GGT GTT CAT	GCT GGC AGA TCA TCG AGT T	/56-FAM/CC TTG GCC A/Zen/C ACC GAC ATC GAT /3IABkFQ/
vdh	GTG GAC GGG AAA GAT CCA T	CGA TCT CGG CGA TCT GTT	/56-FAM/TG GGA CCA C/Zen/G AAG GTC ACC AAC /3IABkFQ/
vdh2	ACG TGC GGA ATG CTA TGA	GAC GCG AGA ACG ACC AG	/56-FAM/AC GGA TGT G/Zen/T CGG AAG ACC TGC /3IABkFQ/
gcoA	GGA CGT CAG TTC GGA CAA A	GAA ACG TTC CTG GTT CAG AAA G	/56-FAM/CT GCG CGA A/Zen/T GGT TCC ACA AAC TG/3IABkFQ/
apkC	ATC GAG GTG ATG AAG CTG ATG	GTA TTC GAC CTG CTC GTT GT	/56-FAM/AC CCT GAC C/Zen/G AGA TCC TCG TCA AG/3IABkFQ/
***sigA*[1]**	GGG TTA CAA GTT CTC CAC CTA C	TTG ATG ACC TCG ACC ATG TG	/56-FAM/TG GTG GAT C/Zen/C GTC AGG CCA T/3IABkFQ/

[1] Reference gene target

Supplementary Table 2: The assembly statistics for GD01 and GD02 are compared to reference genomes.

		Genome			
		**GD01**	**GD02**	**EP4**	**RHA1**
**Assembly Statistics**	Length (Mb)	6.35	6.29	5.72	9.7
	CDS	5860	5768	4976	8446
	GC (%)	67.75	67.79	67	66.97
	Contigs	32	24	2	4
	Scaffolds	32	24	1	4
	N50 (Mb)	0.99	1.81	5.56	7.8
	L50	3	2	1	1
**Completeness**	CheckM	99.4	99.2	99.4	99.6
	BUSCO	99.2	99.2	99.2	99.2

Supplementary Table 3: Annotation of GD01 and GD02 genes involved in the whole putative pathway to degrade BL monoaromatics.

Gene	Description	GD01 Gene ID	GD02 Gene ID	Ref Strain	Accession No	GD01 %ID	GD02 %ID	Ref
catA	catechol 1,2-dioxygenase	2_977	3_1052	RHA1	WP_009475036.1	69.04	69.00	(Patrauchan et al., 2005)
catB	muconate cycloisomerase	2_976	3_1051	RHA1	WP_009475035.1	75.47	75.74	(Patrauchan et al., 2005)
catC	muconolactone D-isomerase	2_975	3_1050	RHA1	WP_009475034.1	90.32	90.32	(Patrauchan et al., 2005)
pcaB	3-carboxy-cis,cis-muconate cycloisomerase	2_951	3_1017	RHA1	WP_011594388.1	59.26	59.49	(Patrauchan et al., 2005)
pcaG	protocatechuate 3,4-dioxygenase, α subunit	4_413	1_409	RHA1	WP_011594387.1	40.58	40.58	(Patrauchan et al., 2005)
pcaH	protocatechuate 3,4-dioxygenase, β subunit	4_412	1_410	RHA1	WP_009474041.1	48.26	48.70	(Patrauchan et al., 2005)
pcaI	3-oxoadipate CoA-transferase, α subunit	2_950	3_1016	RHA1	WP_005248111.1	80.65	80.65	(Patrauchan et al., 2005)
pcaJ	3-oxoadipate CoA-transferase, β subunit	2_949	3_1015	RHA1	WP_009474040.1	76.70	76.70	(Patrauchan et al., 2005)
pcaL	3-oxoadipate enol-lactonase	2_952	3_1018	RHA1	WP_011594389.1	63.57	63.05	(Patrauchan et al., 2005)
vanA	vanillate *O*-demethylase, oxygenase	2_967	3_1042	RHA1	WP_011596645.1	35.41	30.10	(Chen et al., 2012)
vanB	vanillate *O*-demethylase, reductase	2_968	3_1043	RHA1	WP_011596643.1	39.78	38.95	(Chen et al., 2012)
vdh	vanillin dehydrogenase	3_422	2_1324	RHA1	WP_011595659.1	53.29	52.40	(Chen et al., 2012)
gcoA	guaiacol *O*-demethylase, cytochrome P450	2_984	3_1059	RHA1	WP_011595125.1	76.43	76.40	(Fetherolf et al., 2020)
gcoB	aromatic O-demethylase, reductase	2_985	3_1060	RHA1	WP_011595126.1	59.70	59.70	(Fetherolf et al., 2020)
apkC	putative alkyl-phenyl ketone carboxylase, γ subunit	32_2	13_4	*Aromatoleum aromaticum* EbN1	CAI06288.1	41.10	46.50	(Wohlbrand et al., 2008)
apkB	putative alkyl-phenyl ketone carboxylase, β subunit	32_3	13_5	*Aromatoleum aromaticum* EbN1	CAI06287.1	50.22	50.20	(Wohlbrand et al., 2008)
apkA	putative alkyl-phenyl ketone carboxylase, α subunit	32_4	13_6	*Aromatoleum aromaticum* EbN1	CAI06286.1	44.82	44.80	(Wohlbrand et al., 2008)

3 Supplementary Figures

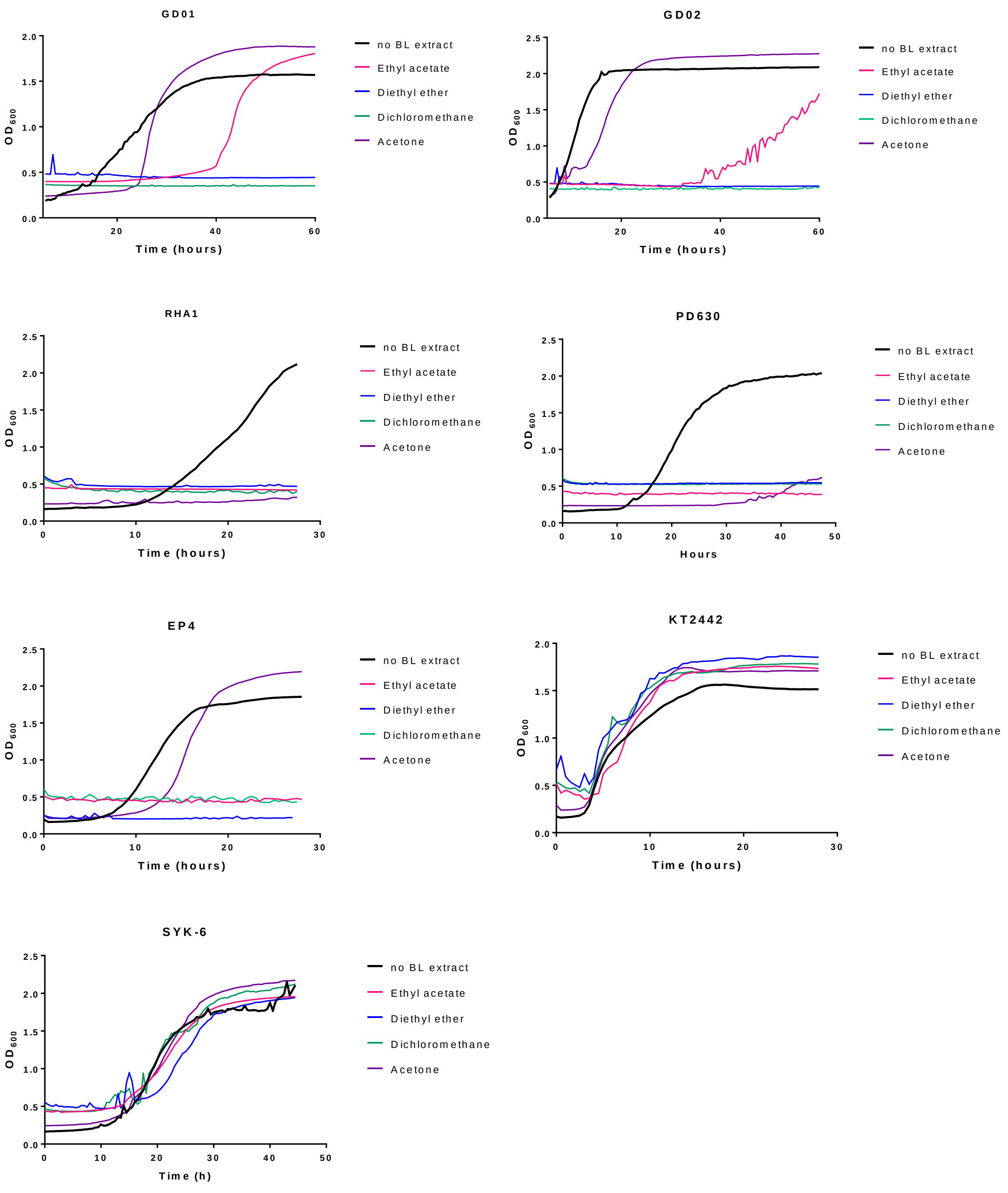

Supplementary Figure 1. Inhibition of strain growth on LB by different solvent extractions of black liquor diluted to 1 mM total aromatic compounds. Growth was monitored continuously at 30 °C using a plate reader. Curves represent single replicates that are representative of triplicate experiments.

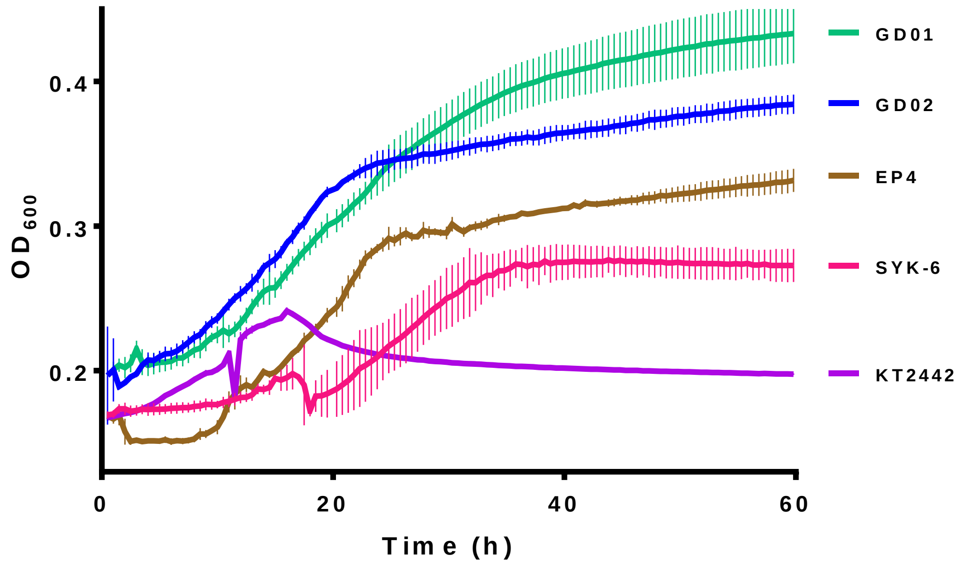

Supplementary Figure 2. Growth of bacterial strains on a mixture of the black liquor aromatic compounds. Strains were grown at 30 °C on a mixture of six aromatic compounds in the same proportion as in the BL acetone extract in defined medium, total concentration 2 mM. OD_{600} was continuously monitored using a plate reader. Solid lines represent the average of three replicates. The vertical bars indicate the standard deviation.

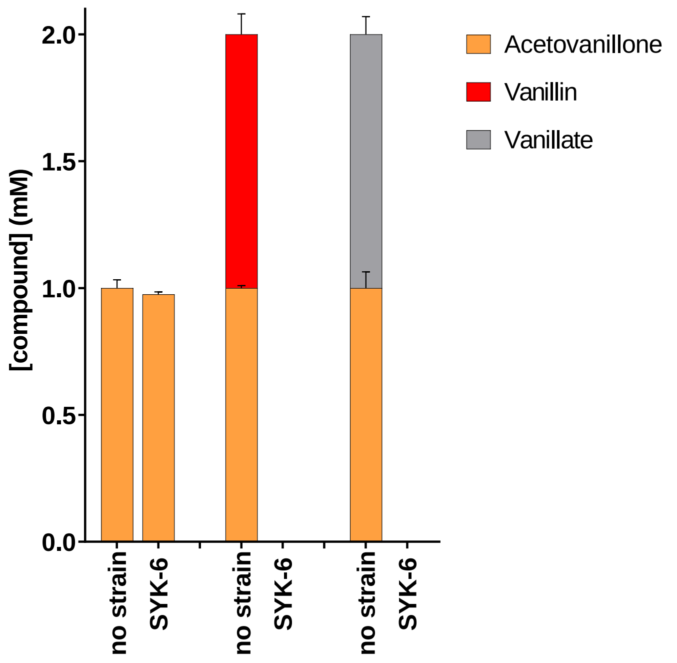

Supplementary Figure 3. Depletion of acetovanillone by SYK-6. SYK-6 was incubated for 96 h on minimal medium amended with 1 mM acetovanillone in presence and absence of 1 mM vanillin or vanillate. Controls with no strain were performed in parallel. Compounds were quantified using GC-MS.

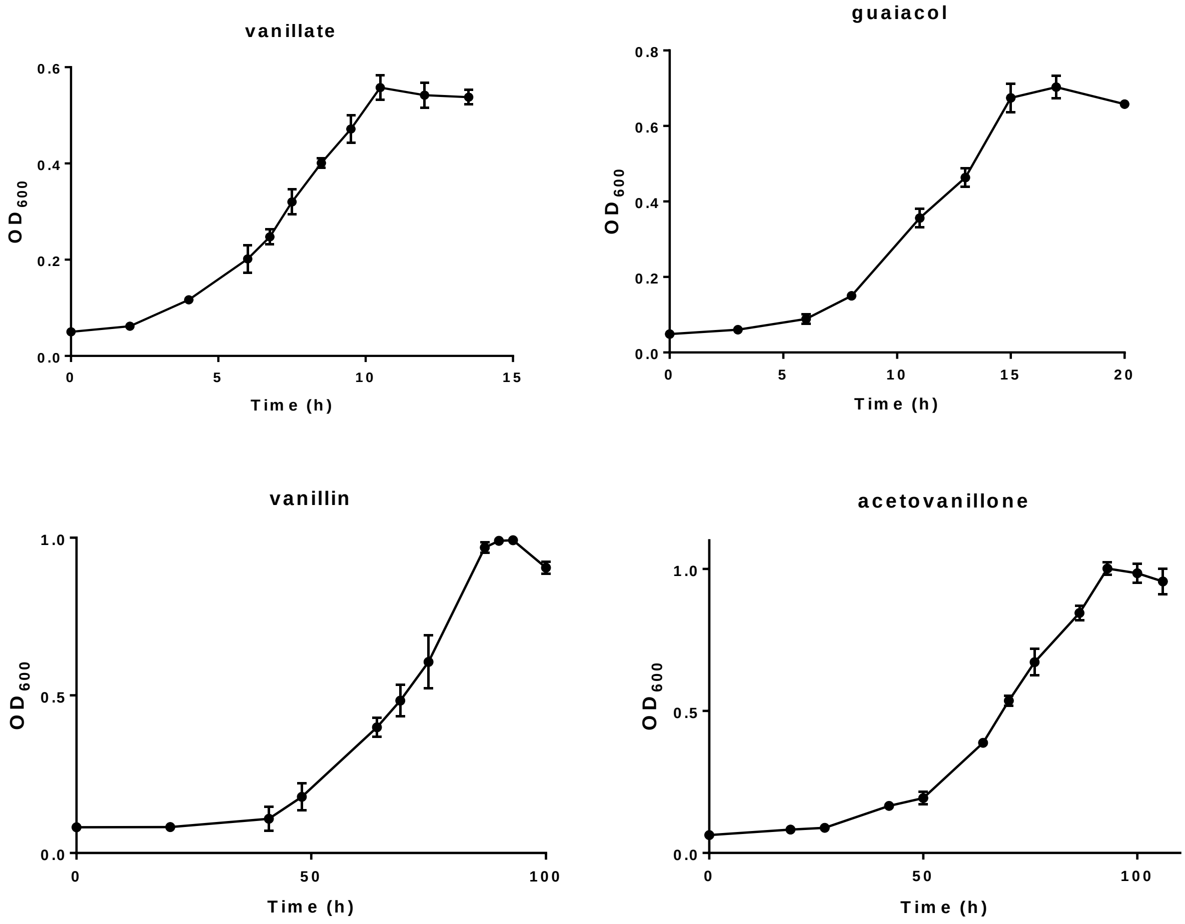

Supplementary Figure 4. Growth of GD02 on select aromatic compounds. GD02 was grown in shake flasks at 30 °C on minimal medium amended with 2 mM of the indicated compound. Data points represent the average of three replicates, the vertical bars indicate the standard deviation.

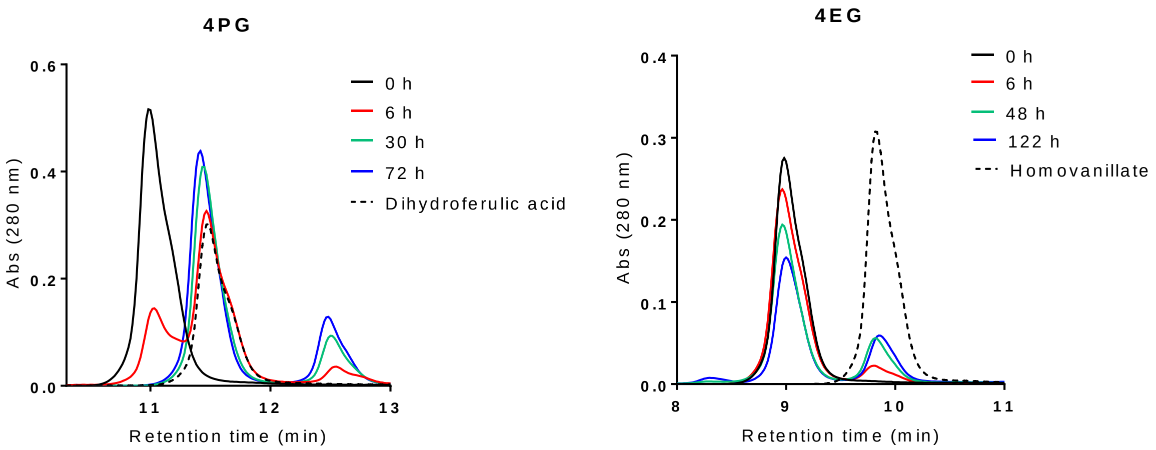

Supplementary Figure 5. Transformation of 4PG and 4EG by GD02. Cells were grown on 1 mM mixture of monoaromatics in the same proportion as in the BL acetone extract, harvested to mid-log phase, suspended in M9 media containing 10 mM 4PG or 4EG, and incubated at 30 °C. Aliquots were withdrawn at the indicated times, and supernatants were analyzed using HPLC.
